# Ultrapure Water Production by a Saline Industrial Effluent Treatment

**DOI:** 10.3390/membranes15040116

**Published:** 2025-04-07

**Authors:** Adriana Hernández Miraflores, Karina Hernández Gómez, Claudia Muro, María Claudia Delgado Hernández, Vianney Díaz Blancas, Jesús Álvarez Sánchez, German Eduardo Devora Isordia

**Affiliations:** 1 Tecnológico Nacional de México, Instituto Tecnológico de Toluca, Av. Tecnológico S/N, Col. Agrícola Bellavista, Metepec 52149, Mexico; dd2281236@toluca.tecnm.mx (A.H.M.); khernandezg@toluca.tecnm.mx (K.H.G.); mdelgadoh@toluca.tecnm.mx (M.C.D.H.); vianney.db@toluca.tecnm.mx (V.D.B.); 2 Departamento de Ciencias del Agua y Medio Ambiente, Instituto Tecnológico de Sonora, 5 de febrero 818 sur Col. Centro, Cd Obregón 85000, Mexico; jesus.alvarez@itson.edu.mx (J.Á.S.); german.devora@itson.edu.mx (G.E.D.I.)

**Keywords:** desalination, reverse osmosis, water recovery, ion exchange resins, antiscalant, deionized water

## Abstract

A membrane system was applied for ultrapure water production from the treatment of saline effluent from the canned food industry. The industrial effluent presented a high saline concentration, including sodium chloride, calcium carbonate, calcium sulfates, and magnesium. The effluent was treated using a system of reverse osmosis (RO) and a post-treatment process consisting of ion exchange resins (IEXRs). The RO was accompanied by the addition of a hexametaphosphate dose (2, 6, and 10 mg/L) as an antiscalant to avoid the RO membrane scaling by minerals. In turn, IEXRs were used for water deionization to produce ultrapure water with a reduced concentration of monovalent ions. The antiscalant dose was 6 mg/L, producing clean water from RO permeates with an efficiency of 65–70%. The brine from RO was projected for its reuse in food industry processes. The clean water quality from RO showed 20% total dissolved solids (TDS) removal (equivalent to salts). The antiscalant inhibited the formation of calcium salt incrustation > 200 mg/L, showing low fouling. In turn, anionic resins removed 99.8% of chloride ions, whereas the monovalent salts were removed by a mix of cationic–anionic resin, producing ultrapure water with electrical conductivity < 3.3 µS/cm. The cost of ultrapure water production was 2.62 USD/m^3^.

## 1. Introduction

Currently, the environmental problems of water scarcity and contamination have changed the perspective of wastewater treatments toward a sustainable approach, involving water and component recovery for recycling. In this regard, diverse industrial effluents can be treated for water reuse. In particular, saline industrial effluents could be purified to produce clean water for different applications, including production areas [1]. However, water recovery from industrial effluents requires an adequate system of treatment to achieve the water recycling objectives [2].

The commercial process of desalination involves membrane technologies, such as reverse osmosis (RO) and electrodialysis (ED), and thermic desalination, such as multiple effect distillation (MED) and multi-stage flash (MSF). Also, there are other technologies used for water desalination, including membrane distillation (MD), forward osmosis (FO), capacitive deionization (CDI), and humidification dehumidification (HDH); however, they are all still under investigation.

Currently, the most used desalination process is RO, with 85% of the desalination plants across the world using RO because this technology presents a high desalination rate and the energy required is less than for thermic operations. However, RO also has several challenges, such as diminishing membrane fouling and reducing the energy demand [3].

Membrane fouling is caused by the presence of colloidal, organic, and inorganic particulate and or biofouling. In particular, the presence of calcium, sodium carbonates, and sulfates produces fouling in scaling form with mineral deposits on the membrane surface. In addition, the scaling is increased by concentration polarization near the membrane surface via incomplete salt dilution [4,5,6].

In any form, fouling phenomena affect the efficiency of the desalination process in terms of water permeation, salt rejection, the quality of the recovered water, and the costs of inversion and operation.

The reduction in membrane fouling has been addressed using various alternatives. Currently, there are available reports on water desalination using membranes with high effectiveness for salt rejection and a high capacity to diminish salt deposition on the membrane surface [7]. However, the membrane market is limited, and there are few options for membrane material selection. Another alternative is the use of antiscalants to decrease the salt concentration on the membrane surface and the precipitation of minerals. Antiscalants are inhibitors or dispersants of sulphate and carbonate salts that reduce calcium, magnesium, and sodium deposition; their use and dose should be studied to achieve optimum results [6,8].

Previous reports by Khalek et al. [9], De Morais et al. [10], and Hasson et al. [11] have shown the use of sodium hexametaphosphate (SHMP) as an antiscalant to inhibit calcium salt incrustation during water desalination using an RO membrane. Yerzhan et al. [12] used antiscalant amino phosphonic acid for CaCO_3_ and CaSO_4_ removal, whereas Pramanik et al. [13] added polyaspartic acid to enhance the desalination treatment, increasing the water recovery. Shen et al. [14] used polyacrylic acid, while Ramírez et al. [15] applied poly(methyl) vinyl ether-alt-maleic acid and D-gluconic acid as an antiscalant for NaCl and Na_2_SO_4_, enhancing the water permeation.

In turn, ultrapure water purification from industrial effluent treatment has superior quality requirements to refine reuse, which allows RO post-treatment processes to complement the effluent depuration.

Ultrapure water production involves water deionization. Ions are removed via ion exchange operations, reducing dissolved salts and minerals from water.

Deionization requires the use of material beds with a high capacity for ion exchange and easy recuperation. The most effective materials are resins from an ion exchange. Ion removal is carried out by attracting and trapping oppositely charged ions [16].

Water deionization processes comprise single-ion exchange (IEX) and electrodeionization (EIEX) (the combination of electrodialysis (EDI) with an ion exchange). The use of IEX for water preparation can be seen in the investigation of Alvarado and Chen [16]. The authors produced ultrapure water for water reuse in power plants. Song et al. [17] studied the production of ultrapure water from RO permeates via the EIEX process to achieve 98% to 99% salt removal and cationic ions. Anionic ion removal, such as Cl^−^ and nitrate, can also be seen in other investigations [18,19,20,21].

According to the latest studies, ultrapure water production by saline industrial effluent treatment requires the design of an integrated or hybrid system for water production, which depends on the quality of the water application.

In this investigation, we integrated a hybrid process of desalination and deionization of a food industrial effluent for water recovery, producing ultrapure water.

RO was applied for effluent desalination, using an antiscalant to reduce the scaling in the membrane and increase the efficiency of water production.

In turn, an IEX process was tested as post-treatment to RO to provide ultrapure water for industrial operation use.

The data obtained on the integrated RO-IEXR system for desalination and deionization are useful in the design of this process at the industrial level.

## 2. Materials and Methods

### 2.1. Reagents and Solutions

The antiscalant sodium hexametaphosphate was supplied by CTR Scientific, (Monterrey, Mexico), with a purity of 98.2%.

### 2.2. Food Effluent Valorization to Treatment and Water Recovery

The effluent was supplied by the food production industry (10 L) and stored in flasks of 1 L at 4 °C for characterization and treatment.

The industrial effluent was valorized for its treatment as raw effluent or diluted effluent.

The physicochemical characteristics of effluents were determined using standard methods for the examination of Water and Wastewater (APHA 2023) [22], including pH, conductivity (EC), total dissolved solids (TDS), total solids (TS), and total suspended solids (TSS). Ions in effluent Cl^−^, Na^+^, and Ca^2^ were measured potentiometrically using a HANNA multiparameter HI3221-01 (Woonsocket, Rl, USA) with ion-selective electrodes. Mg^2+^ was measured by volumetric titration, and ions of SO_4_^2−^ were measured using the turbidity method.

### 2.3. Desalination Technology for Industrial Effluent Treatment

The desalination technology consisted of a pilot system of reverse osmosis, using a polyamide membrane with a permeation area of 0.0491 m^2^. The operating conditions were determined by membrane function, using deionized water as the feed. The pressure range was 8–9 bar and the permeate flow was 5–8 L/h at ambient temperatures (20–22 °C). The hydraulic resistance of the RO membrane and the water permeability coefficient were 4.6 × 10^−5^/m and 0.15 L/h/m^2^ bar, respectively.

### 2.4. Water Recovery from Food Effluent Desalination Process

The experimental desalination of industrial effluent was applied to raw effluent and diluted effluent (35%), each with antiscalant Sodium-Hexametaphosphate (SHMP) doses of 0, 2, 6, and 10 mg/L. The SHMP dose was previously established according to the TDS concentration in the effluent.

The effluents were fed into the RO system at a continuous flow, operating with a transmembrane pressure < 2 bar and input and output pressures of 7–9 bar.

The desalination data were collected using an experimental design to determine the impact of the TDS concentration in the effluent and the SHMP dose on the membrane flux performance. The collected data were averaged from at least 5 samples for the effluent and SHMP dose.

The performances of the reverse osmosis membranes were obtained using the following equations:

(1) The permeate flux density (J_p_) (L/min/m^2^) was given by Equation (1), where *Q_p_* is the permeate flow (L/h) and *A* is the active area of the membrane in m^2^.

(1) $$J_{p}=\frac{Q_{p}}{A_{m}}$$

(2) The percentage of water recovery (R%) by the membrane was calculated using Equation (2), where Q_p_ is the permeate flow and Q_a_ is the feed flow given in mL/min.

(2) $$R\%=\frac{Q_{p}\;*\;100}{Q_{a}}$$

(3) The salt rejection percentage (SR%), given as a function of TDS rejection% or ion rejection percentage (IR%) as Ca^2+^, SO_4_^2−^, Na^1+^, and Cl^1−^% removal, was calculated using Equations (3) and (4), where C_f_ is the concentration of TDS or ions in the feed and C_p_ is the concentration of TDS or ions in the permeate.

(3) $$SR\%=\frac{\left(C_{f}-C_{p}\right)100}{C_{f}}$$

(4) $$IR\%=\frac{\left(C_{f}-C_{p}\right)100}{C_{f}}$$

(4) Salts permeation percentage (SP%) given as function of TDS% permeation, or ions permeation percentage (IP%) as Ca^2+^, SO_4_^2−^, Na^1+^ and Cl^1−^%, using Equation (5). Where C_p_ is the concentration of TDS or ions in the permeate.

SP% = 100 − IR% or IP% = 100 − IR%(5)

(5) The percentage and fouling index of the membrane (F% and SDI) were calculated using Equations (6) and (7), where t_15 min_ is the operation time for the membrane to fill 25 mL in a test tube after 15 min of the desalination process, t_i_ is the initial operation time, and t is time elapsed between the initial measurement and the second measurement.

(6) $$F\%=\frac{\left(t_{15\;min}-t_{i}\right)*100}{t_{15\;min}}$$

(7) $$SDI=\frac{F\%}{t}$$

(6) The hydraulic resistance of the membrane (HRM) is given by Equation (8), where ∆P is the pressure difference at the outlet and inlet of the system when the water or effluent is fed, μ the viscosity of the clean water or the effluent, and J_p_ the permeate flux density during the RO process.

(8) $$HRM=\frac{∆P}{\mu J_{p}}$$

(7) The hydraulic permeability coefficient (L_p_) is calculated with Equation (9), where J_p_ is the permeate flux density and TMP is the transmembrane pressure.

(9) $$L_{p}=\frac{J_{p}}{TMP}$$

(8) Scanning electron microscopy (SEM) of the membrane surface morphology was applied after membrane use in the desalination process and cleaning to verify the membrane integration. SEM was obtained using a scanning electron microscope from the JEOL JSM-6610LV series (JEOL company, Akishima, Tokyo, Japan).

### 2.5. Ultrapure Water Production from RO Stream

According to the requirements of water reuse in industry, treatment by RO included post-treatment consisting of water deionization for ultrapure water production using the IEX process with ionic resins to reduce the remnant ions.

In this step, different ionic resins were tested for water deionization independent of ion content. The resins were rinsed with deionized water to expand their volume for 24 h. Subsequently, each resin was packed in a glass column with dimensions of 34 mm in diameter and 450 mm in length. Column packing was performed using a bed with a volume of 40 mL. Once the columns were packed and prepared, they were run at a feed flow of 10 mL/min. The data on ion removal and curves of the break were collected to compare the resin’s effectiveness.

A cost analysis of ultrapure water production via the operational process was introduced to analyze the treatment feasibility of industrial effluent.

## 3. Results

### 3.1. Physicochemical Characteristics of Industrial Effluent

Table 1 shows the physicochemical characteristics of the raw and diluted effluent.

The data display basic pH and saline properties of effluent, such as high EC and TDS. The effluent did not show SS and TSS; however, they exhibited similar values of TDS and TS. In turn, the diluted effluent showed reduced values compared to the raw effluent, so we decreased the salinity to test the best conditions of effluent for its treatment.

The ion concentration in the raw and diluted effluents indicated a high content of Ca^2+^, SO_4_^2−^, Cl^−^, and Mg^2+^, which were associated with a high possibility of membrane fouling due to salt precipitation on the membrane surface. In addition, the ion concentration was specified in the characteristics of the effluent because the recovered water requires water deionization for its reuse.

### 3.2. Water Recovery Efficiency from Effluent Desalination Process

The water recovery efficiency by RO effluent desalination (raw and diluted 35%) using SHMP doses of 0–10 mg/L is presented under the following conditions: (1) TDS% removal in clean water permeates from RO, including ion concentration % (Ca^2+^, Cl^−^, and others); and (2) functional parameters of RO such as clean water flux (J_p_%), salt rejection (SR%), membrane fouling (F%), and the Silt Density Index (SDI). The recollected data correspond to the average of three samples of raw effluent and diluted effluent with three replicates.

Table 2 shows that TDS removal in raw effluent by RO without an SHMP dose was 50%, whereas the diluted effluent showed 70% TDS removal. The ion removal ranges were 60–70% in raw effluent and 50–80% in diluted effluent. In particular, the removal of Ca^2+^ ions was 60% and 65% for raw and diluted effluents, respectively. Therefore, effluent dilution enhanced TDS and ion removal by 8–10%.

In turn, SHMP addition increased the percentage of TDS and ion removal from raw and diluted effluent, showing significative differences according to the dose. An SHMP dose of 6 mg/L caused the highest increases in TDS and ion removal, showing 66 and 75% of TDS, whereas Ca^2+^ ion removal showed 70 and 90% for raw and diluted effluents, respectively. In this case, SHMP enhanced TDS removal by 20%; however, the best result was observed in Ca^2+^ ion removal, avoiding scale deposition in the membrane (>200 mg/L). 

An SHMP dose of 6 mg/L also increased the ion removal of Mg^2+^, achieving 80% in the raw effluent and 90% in the diluted effluent. However, 35% of Cl^−^ ions were removed; thus, remnant Cl^−^ ions and remnant salt were detected in the permeates of RO.

The scaling formation has been described by two fouling mechanisms [23]: (1) bulk crystallization and deposition or heterogeneous surface crystallization, and (2) surface crystallization.

Fouling from bulk crystallization is caused by crossflow velocities and higher operating pressures, whereas surface crystallization presents with a higher operating pressure and a low flow velocity. This type of scaling was attributed to concentration polarization [24].

In turn, the fouling removal mechanism of calcium and magnesium ions by SHMP was based on the sequestrating of ions to form stable and insoluble complexes in the water, reducing their precipitation on the RO membrane. 

**Figure 1:**
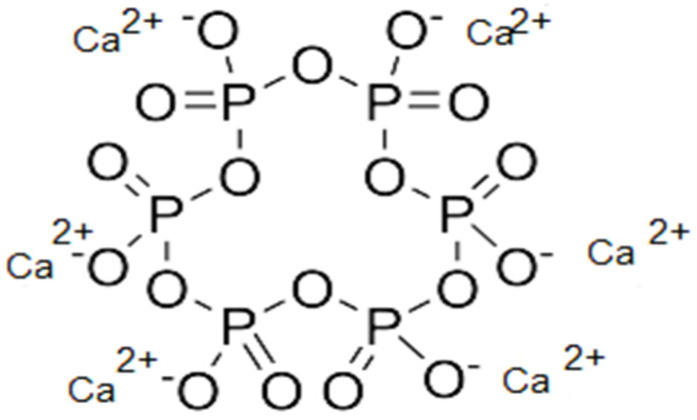
Complexation mechanism of SHMP with calcium ion.

Figure 1 shows the SHMP complexation with calcium ions, suggesting the inhibition of calcium carbonate scaling. 

A similar mechanism has been suggested for magnesium complexation, where the calcium and magnesium ions present in the water are captured by phosphate groups on SHMP [25].

The neutral and alkaline pH (8.5) of effluent aids in calcium sequestering by SHMP because the predominant species of calcium carbonate is HCO_3_^−^, whereas the predominant species of SHMP are H_4_(PO_3_)_6_^2−^ and H_2_(PO_3_)_6_^4−^, which are more energetically favorable for capturing free calcium ions and more possibilities of interactions between SHMP and calcium because the number of active scale sites of SHMP increases [6,23,25].

Furthermore, SHMP decreased the surface tension of the water, improving membrane permeation. Table 3 exhibits the information on RO membrane efficiency during the desalination of industrial effluents (raw and diluted 35%) with different doses of the SHMP antiscalant.

The data show that an SHMP dose of 6 mg/L in the raw effluent also produced increases in fluxes J_p_ (5%) and salt rejection (SR = 10–20%), while the diluted effluent with 6 mg/L of SHMP achieved a 15% increase in J_p_ and a 30–35% reduction in fouling.

Consequently, reversible fouling was reduced with an SHMP dose of 6 mg/L. The raw effluent showed 85% fouling, whereas the diluted effluent showed 35%. Similar data were provided by 6 mg/L of SHMP on SDI, reducing SDI by 30–40% in raw and diluted effluents, respectively. However, an SHMP dose > 6 mg/L increased the fouling of the RO membrane, while a 2 mg/L dose of SHMP did not display a significative effect on desalination efficiency (*p* < 0.05).

The latest information is complemented by the kinetic of RO permeates’ behavior during the desalination of industrial effluent (raw and diluted) using SHMP as an antiscalant in doses of 0–10 mg/L. 

**Figure 2:**
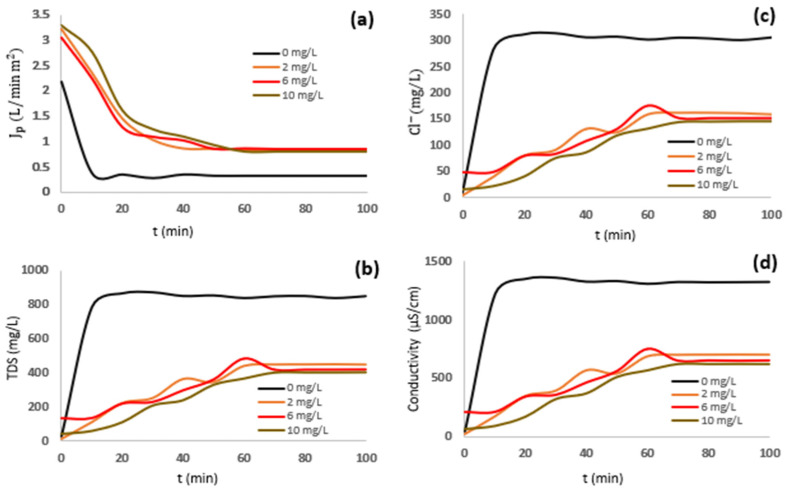
RO membrane permeates during desalination of raw effluent, using different doses of SHMP. (**a**) Water flux, (**b**) TDS, (**c**) chlorides, and (**d**) conductivity.

**Figure 3:**
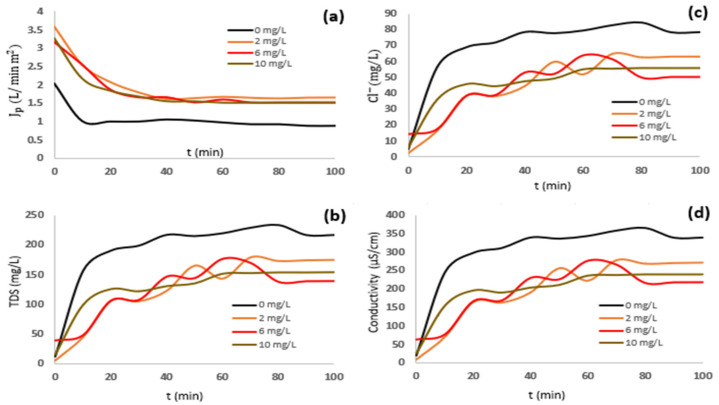
RO membrane permeates during desalination of diluted effluent (35%), using different doses of SHMP. (**a**) Water flux, (**b**) TDS, (**c**) chlorides, and (**d**) conductivity.

Figure 2 and Figure 3 show the RO permeates’ behavior, including flux, TDS, EC, and Cl^−^ removal.

The diluted effluent with 6 mg/L of the SHMP antiscalant showed an increase in J_p_ of 50% and a 30% reduction in fouling. Positive ion removal achieved 90%, whereas Cl^−^ and SO_4_^2−^ ions achieved 70 and 90% exclusion. Therefore, the best conditions for the RO process were suggested to be the use of diluted effluent with an antiscalant dose of 6 mg/L because the RO membrane achieved the highest water flux and Cl^−^ removal, enhancing the membrane life, with 70% Cl^−^ removed by the membrane. In addition, the scaling dominating the fouling of the membrane was reduced due to the complexation of SHMP with ions, calcium, sodium, magnesium, and sulphates, achieving a satisfactory SDI index. Consequently, an SHMP dose > 6 mg/L may reduce the particle size of the precipitate and cause increases in fouling.

**Figure 4:**
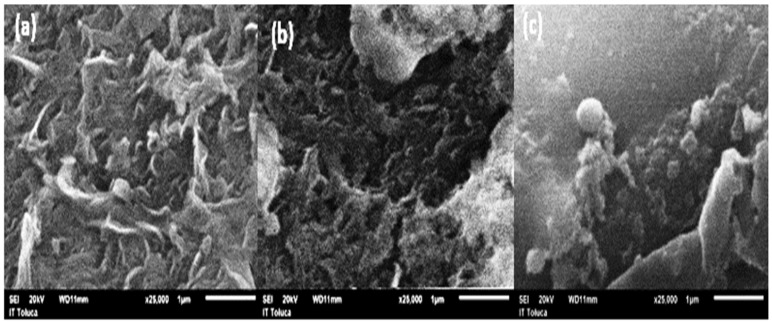
SEM images of membrane surface of polyamide membrane of RO. (**a**) Before of the effluent desalination. (**b**) After of 3 h of effluent desalination, using SHMP. (**c**) After effluent desalination without SHMP. Resolution ×25,000.

In turn, Figure 4 shows the SEM images of the RO surface membrane morphology (a) after its use in the desalination process with 6 mg/L of SHMP, (b) after its use in the desalination process without SHMP, and (c) with a clean membrane of polyamide.

The SEM images in Figure 4b,c show (a) polyamide membrane before of effluent desalination. (b) the polyamide membrane after of 3 h of desalination, using SHMP. (c) the polyamide membrane after 3 h of desalination without antiscalant. Here, it is evident that after 3 h of effluent desalination, the salt deposition on the membrane surface was greater when the operation was carried out without SHMP than the desalination using SHMP, which demonstrated less fouling.

Previous studies on antiscalant use have shown that SHMP is an effective antiscalant in membrane operation; however, the dose should be studied because it is dependent on the salt content in feed water. The SHMP could also cause contrasting effects such as salt precipitation, turbidity, and limitations in membrane operation [6].

Some studies showing the use of antiscalants in RO can be found in Morais et al. [10]. The authors found that an antiscalant dose of SHMP of 2.5 mg/L achieved the complete inhibition of calcium carbonate in the process of water desalination. However, Khalek et al. [9] showed that the optimal concentration range of SHMP was >5 mg/L to prevent membrane fouling by calcium precipitation.

In turn, Pramanik et al. [13] showed that the use of a high dose of the antiscalant poly-aspartic acid increased the salt rejection percentage (93.4%), enhancing the conductivity in membrane permeates during the desalination of brackish water.

### 3.3. Production of Ultrapure Water

Accordingly, the presence of Cl^−^ ions and remnant ions in the permeates of RO suggested that the post-treatment process for ion reduction, with principal emphasis on Cl^−^ removal, can enhance water quality. Consequently, TDS and EC were also diminished in the production of ultrapure water, ensuring its recycling in industrial activities.

Table 4 shows the data on water treatment by IEX, including water dichlorination, using anionic resins IEXR1 or IEXR2 as the first step and IEXR3 (mixed resin) as the second step for remnant ion removal (cations and anions).

IERX1 showed 60% efficacy in dichlorination, whereas IEXR2 was superior, displaying 80%. Therefore, IEXR2 was selected in the first step of treatment as the more effective resin. In the second step of deionization, IEXR3 exhibited remnant ion removal, achieving ultrapure water quality.

The efficiency of the IEXR2 treatment was attributed to the type of resins, acting as an anionic base supported by a polymeric matrix called styrene-divinylbenzene, whose use consists of removing anions such as Cl^−^ from water [26,27]. IEXR3 corresponds to a mixture of strong acid cationic and strong base anionic resins supported on a gel-type styrene-divinylbenzene polymeric matrix, which is used to remove both anions and cations from water. In addition, the feed flow and concentration of salts in the feed contributed to the IEXR treatment efficiency.

**Figure 5:**
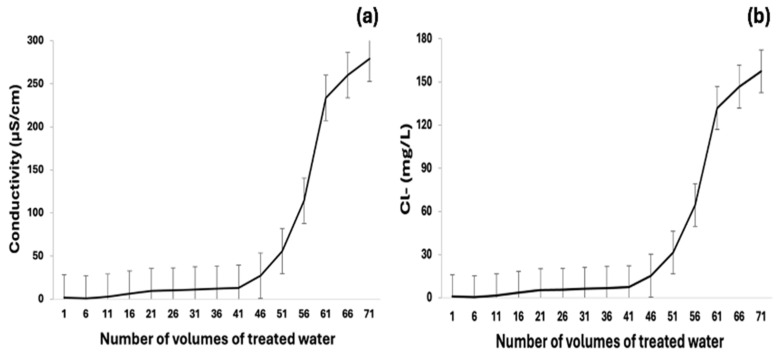
Break curves of ions removal in water from RO, using IEXR3. (**a**) Conductivity of treated water during deionization. (**b**) Cl ions of treated water during dichlorination.

In turn, Figure 5 shows the efficiency behavior of IEXR3 (mixed resin), showing break curves of treated water, including Cl^−^ and EC values versus volumes (V) of water treatment, where each volume corresponds to 40 mL.

The break curves show that a bed of IEXR3 (40 mL) has the capacity to treat 1.7 L of water from the RO, during 43 times without altering the properties of the deionized water and the IEXR3. However, after 43 volumes of water deionization, the values of Cl^−^ and EC are increased, indicating the resin saturation and cycles of resin washing and regeneration required in the process.

According to the latest information, the global efficiency of treatment processes of ultrapure water from industrial effluents is adequate, achieving the objective of desalination and deionization with a high efficiency of 99.9%.

The preceding information on the use of IEX in water dichlorination and water deionization is found in previous publications, showing similarities with this report; however, the data are isolated and different because the coupled processes are diverse and the water feed and water requirements of purification and reuse correspond to water recycling. For example, Boonpanaid et al. [27] reported the use of cationic resins for calcium and magnesium removal, achieving 45% efficiency. In this case, the feed water was domestic with a low salt content of 109.8 ± 20.56 mg/L.

Li et al. [28] also used IEX for the elimination of calcium and magnesium, showing a greater affinity for calcium with multiple period cycles of removal.

Khoiruddin et al. [29] examined the performance of EIEX for the desalination of brackish water, showing 99% TDS removal and 75% water recovery.

Otero et al. [30] used an EIEX process for salt removal in saline water. In this case, the authors reached 73.34% NaCl from a concentration of 0.01 M of NaCl.

In turn, Rawajfeh [31] tested Cl^−^ ion removal from water using a pre-electrode adsorption filter of the EIEX unit with feldspar, Tripoli, pozzolana, and Mg-Al layered double hydroxide (LDH) materials, achieving 60% chloride ion removal.

### 3.4. Integrated System of RO for Ultrapure Water Production from Saline Industrial Effluent

Matching the previous results, ultrapure water production from saline industrial effluent required a coupled system of RO, consisting of one step of RO and two steps of deionization using the IEX process. The use of the antiscalant SHMP in RO enhanced membrane permeation, increasing the efficiency in clean water recovery and fouling reduction by 20%. The first step of deionization consisted of water dichlorination, resulting in IEXR2 being the best resin for this purpose. The second step was conducted to achieve the quality of ultrapure water characteristics by IEXR3, removing remnant Cl^−^ and Na^+^ ions.

**Figure 6:**
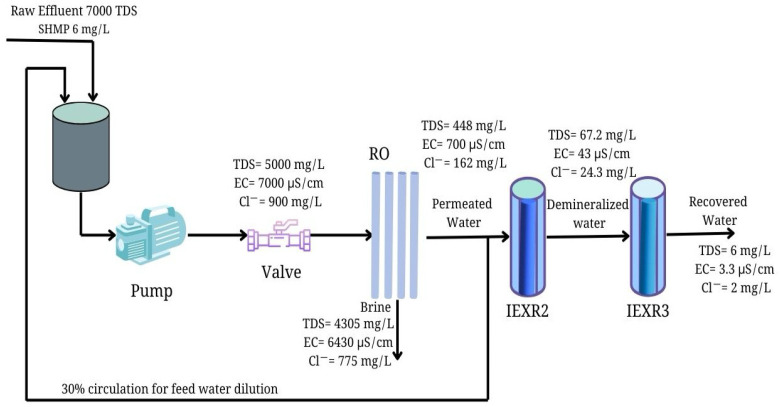
Process diagram of RO system for water recovery from industrial effluent treatment and data on water quality.

In accordance with the data on industrial effluent treatment, Figure 6 displays a process diagram of the RO system for industrial effluent treatment for water recovery.

The suggested system of industrial effluent treatment includes an RO process for water desalination using the antiscalant SHMP. In turn, IEX processes are coupled to RO to produce ultrapure water via ion removal, using two steps of deionization with a principal focus on Cl^−^ and Na^+^ reductions. The RO system indicates that 70% of recovered water is directed for reuse, whereas 30% is suggested for feed water to achieve 35% dilution in raw industrial effluent.

In turn, Table 5 exhibits the quality characteristics of treated water (TDS, EC, and Cl^−^) according to the treatment step of industrial effluent treatment, including the input and output of streams from the RO system.

In comparison with other studies based on RO industrial effluent treatment, our proposal of the RO system used in this report was analogous to the process offered by Santos et al. [32]. Those authors integrated an RO procedure to produce demineralized water from a petrochemical effluent. However, the RO system included UF, forming a UF-RO-EIEX system. The process showed the capacity of clean water production with a 98% global TDS removal and high-quality characteristics in the recovered water.

In the report by Hernandez et al. [1], the authors described a hybrid membrane process integrated with sedimentation (S), adsorption by activated carbon (AC), ion exchange resins (IEXRs), and RO (S–AC–IEXR–RO) to treat an industrial effluent. Using the RO pretreatment, fouling was reduced and membrane flux increased. The process was efficient in producing clean water, showing rupture points in the curves of AC and IEXR of 5–10 volumes of effluent treatment.

Thu et al. [33] also analyzed the performance of a hybrid system, consisting of ion exchange and reverse osmosis (IEX-RO) for the desalination of brackish groundwater. The IEX operation was used as pretreatment to protect RO membrane fouling. The IEX process included an acid cation exchanger resin for cation removal. IEX removed >250 mg/L of Ca^2+^ and 10,000 mg/L of NaCl. In turn, the authors found high efficiency in the resins (up to 80 bed volumes of water treatment). The efficiency of the RO system was >70%, using an optimized pressure of 120 psi.

Another configuration system of RO for water desalination was reported by Wu et al. [34]. They studied a hybrid system of multi-effect distillation with RO (MED-RO) for the desalination of seawater using mathematical models and software simulation to analyze the influence of feed seawater temperature, salinity, system recovery rate, and membrane element configuration on the performance of the system. The highest recovery rate was 54.22%.

According to the above data, integrated systems of RO for industrial effluent treatment depend on the feed water characteristics and the quality required in the reclaimed water. However, these data could be used for the upscaling of the treatment process of RO.

### 3.5. Operational Costs of Ultrapure Water Production from Saline Industrial Effluent

Table 6 exhibits an approximation of the operational cost of ultrapure water production from saline industrial effluent, resulting in 2.62 USD/m^3^.

According to the operational costs of ultrapure water production, the project could be satisfactory because it is a process of treatment of water recovery from an industrial effluent; therefore, is a sustainable process that addresses the issue of water scarcity. In addition, the treatment of effluent avoids the costs of effluent disposal as wastewater. In this case, the industry spends 2.5 USD/m^3^ on wastewater disposal, which is equivalent to the treatment cost. However, the costs of inversion are required to analyze the global cost of treatment and economic feasibility.

The cost of the treatment process of industrial effluent for ultrapure water production was comparable with previous reports of desalination, although the processes and conditions are different. Mangosing et al. [35] obtained high costs for the treatment of seawater to produce water for domestic use. Those authors used multistage solar desalination technology, and the cost of the project was 9.75 USD/m^3^, indicating an expensive value. The authors concluded that the proposed technology requires development to produce reasonably priced, membrane-based, purified seawater. Triki et al. [36] tested three solar processes to treat a brine effluent, using RO, vacuum membrane distillation (VMD), and an integrated RO-VMD system. The lowest cost of water production was achieved with VMD at 2.16 USD/m^3^, followed by the RO-VMD system at 3.78 USD/m^3^, while RO resulted in 6 USD/m^3^; thus, solar RO required superior energy to treat the effluent.

## 4. Conclusions

Desalination and deionization treatments of saline industrial effluent were applied for water recycling, satisfying the industrial requirements of recovered water with ultrapure characteristics.

According to the salt content, the treatment of industrial effluent was proposed via the use of an RO system consisting of three steps: (1) reverse osmosis (RO) with an antiscalant dose of SHMP of 6 mg/L to enhance the efficiency of RO; (2) ion exchange (IEX) operations using (a) anionic resin for Cl^−^ removal; and (b) mixed resins to complete water deionization.

The antiscalant SHMP enhanced the membrane efficiency, achieving 65% water flux permeation from RO and 90% salt rejection. In turn, TDS decreased by 80% and Ca^2+^ ions were reduced by <200 mg/L; consequently, fouling was reduced due to calcium removal by SHMP. According to the use requirements, ultrapure water was produced by IEX, achieving high-quality characteristics with Cl^−^ ions < 2 mg/L and monovalent salts < 3.3 µS/cm by IEXR2 and IEXR3, respectively.

The last treatment system was designed for treating complex industrial effluent, with the goal of recovering water for reuse as ultrapure water.

The system of treatment integrated RO and IEXR operations to achieve the objective of water quality. Therefore, the suggested system of treatment is dependent on the effluent characteristics and water quality demand.

The treatment system was tested with a commercial additive, membrane, and resins because, for the implementation of the treatment process at an industrial scale, the system should be operationally and technically viable.

The treatment of industrial effluent for ultrapure water production is a sustainable process because ultrapure water production via the treatment of an industrial effluent has a reasonable and competitive operational cost (2.62 USD/m^3^), which is considered satisfactory. In addition, ultrapure water production could address the issue of water scarcity and reduce the costs of water disposal (2.5 USD/m^3^).

Further research should be considered for the scale-up to industrial requirements. In addition, other antiscalants, RO membranes, and IEXR types could be studied to complement this information.

## Figures and Tables

**Table 1:** Physicochemical characteristics of raw industrial effluent and effluent dilution.

Characteristics	Units	Raw Effluent	Effluent Dilution 35%
pH	-	8.5 ± 0.1	7.8 ± 0.1
EC	mS/cm	10 ± 0.1	6.5 ± 0.1
TDS	mg/L	7000 ± 50	5000 ± 50
TS	mg/L	7075 ± 35	5500 ± 15
Cl^−^	mg/L	1530 ± 5	810 ± 10
Ca^2+^	mg/L	516 ± 0.7	450 ± 0.8
Mg^2+^	mg/L	349 ± 0.5	262 ± 0.5
SO_4_^2−^	mg/L	301 ± 0.5	250 ± 0.3

**Table 2:** Data on the percentages of water permeate flux and rejection of quality parameters from industrial effluent treatment (raw and diluted) by RO and different doses of SHMP antiscalant.

Salts Removal Efficiency by RO	Raw Effluent (Initial TDS = 7000 mg/L)				Diluted Effluent (Initial TDS = 5000 mg/L)			
	SHMP Concentration (mg/L)							
	0	2	6	10	0	2	6	10
TDS	50 ± 2	63 ± 5	66 ± 1	77 ± 5	70 ± 1	71 ± 2	76 ± 1	78 ± 3
Ca^2+^	60 ± 1	68 ± 1	70 ± 0.5	71 ± 0.1	65 ± 5	85 ± 0.1	90 ± 5	91 ± 2
Mg^2+^	70 ± 0.5	79 ± 0.3	80 ± 0.5	80 ± 0.1	80 ± 4	90 ± 0.1	90 ± 0.7	90 ± 0.5
SO_4_^2−^	70 ± 0.4	72 ± 0.1	75 ± 0.1	78 ± 0.5	75 ± 2	90 ± 0.5	90 ± 0.5	90 ± 0.3
Cl^−^	60 ± 0.5	65 ± 0.5	69 ± 0.3	70 ± 0.3	50 ± 0.1	78 ± 0.5	79 ± 0.4	79 ± 0.5

**Table 3:** Functional parameters in RO membrane during desalination effluents with SHMP dose.

Functional Parameters in RO	Raw Effluent (Initial TDS = 7000 mg/L)				Diluted Effluent (Initial TDS = 5000 mg/L)			
	SHMP Concentration (mg/L)							
	0	2	6	10	0	2	6	10
J_p_%	50 ± 2	50 ± 1	55 ± 1	55 ± 2	60 ± 4	62 ± 1	65 ± 1	65 ± 2
SR%	50 ± 2	70 ± 5	75 ± 3	75 ± 5	60 ± 1	90 ± 2	93 ± 0.5	93 ± 5
F%	94 ± 1	87 ± 0.5	85 ± 0.5	90 ± 0.2	84 ± 0.5	50 ± 1	35 ± 3	44 ± 1
SDI	6 ± 0.1	4 ± 0.01	4 ± 0.2	5 ± 0.1	4 ± 0.1	2 ± 0.01	3 ± 0.05	3 ± 0.1

**Table 4:** Data on IEXR process application on permeate water from RO membrane, using diluted effluent.

Ionic Resin Comercial Identification/Matrix	Identification/Functional Group	Resins Operation	TDS (mg/L)		EC (µS/cm)		Cl^−^ (mg/L)	
			Input	Output	Input	Output	Input	Output
HPA25 (Strong base anion)/Styrene-DVB, Porous)	EXR1/trimethyl ammonium	First step Dichlorination and anions	448 ± 1	91 ± 7	287 ± 1	58 ± 9	162 ± 1	35 ± 2
WA21J (Weak base anion/Styrene-DVB, Porous)	IEXR2/polyamine	First step Dichlorination and anions	448 ± 2	67 ± 5	287 ± 1	40 ± 5	162 ± 2	24 ± 3
MB3710 (strongly acidic cationic gel and strongly anionic base gel Type I)	IEXR3/Sulfonic Acid and Quaternary Ammonium	Second step Remanant ions (cations and anions)	448 ± 2	6 ± 1	287 ± 1	3.34 ± 1	162 ± 3	2 ± 1

**Table 5:** Quality characteristics of treated water by step of treatment, using RO system.

TDS (mg/L)				EC (µS/cm)				Cl^−^ (mg/L)			
Input RO	Output RO	Output IEX2	Output IEX3	Input RO	Output RO	Output IEX2	Output IEX3	Input RO	Output RO	Output IEX2	Output IEX3
5000 ± 50	448 ± 2	67.2 ± 5	6 ± 1	6500 ± 50	700 ± 5	40 ± 5	3.3 ± 1	810 ± 10	162 ± 3	24.3 ± 3	2 ± 1

**Table 6:** Operational cost of ultrapure water production by industrial effluent treatment.

Operational Parameters	Description	Ultrapure Water Production m^3^/year	Annual Cost of Ultrapure Water Production USD/year	Cost of Water Recovery USD/m^3^
Electric energy	15 kW/h 0.7 USD/kW	87,600	13,140	0.15
Raw Materials	Membranes	87,600	92,820	1.07
	Resins		875	
Chemicals	SHMP	87,600	200	0.2
	Cleaners		300	
Labor operation	Plant manager	87,600	10,800 8400	0.8
	Process engineer			
	Technician		6600	
Maintenance	Salary	87,600	30,500	0.4
Total cost				2.62

## Data Availability

The original contributions presented in this study are included in the article. Further inquiries can be directed to the corresponding author(s).

## Abbreviations

The following abbreviations are used in this manuscript:

A	Cross-sectional area
Cp	Concentration of TDS in the permeate
EC	Electric conductivity
EDI	Electrodialysis
EIEX	Electrodesionization
F%	The percentage of fouling
HRM	The hydraulic resistance of the membra
IEX	Ion exchange
IEXR	Ion exchange resins
IP%	Ion’s permeation percentage
IR%	Ion’s rejection percentage
J_p_	Permeate flux density
L_p_	Hydraulic permeability coefficient
LDH	Layered double hydroxides
∆P	Pressure difference
PTM	Transmembrane pressure
Qa	Feed flow
Qp	Permeate flow
R%	Percentage of water recovery
RO	Inverse osmosis
SDI	Fouling index of the membrane
SEM	Scanning electron microscope
SHMP	Sodium hexametaphosphate
SP%	Salts permeation percentage
SR%	Salts rejection percentage
SS	Suspended solids
TDS	Total dissolved solids
TS	Total solids
TSS	Total suspended solids
t_15 min_	Operation time to fill 25 mL
t_i_	Initial operation time
T	Time elapsed between initial and second measurement.
